# Multicentre randomized controlled trial of acupuncture for vascular cognitive impairment: cognitive benefits and inflammatory biomarker modulation

**DOI:** 10.1016/j.clinsp.2025.100770

**Published:** 2025-09-06

**Authors:** Xixi Han, Baoguo Wang, Lei Xu, Yabin Pi, Fei Li, Tianxin Jiang

**Affiliations:** a Anhui University of Chinese Medicine, China; b The First Hospital Affiliated of Bengbu Medical University, China; c Bozhou Hospital of Traditional Chinese Medicine, Anhui, China; d The second Affiliated Hospital of Anhui University of Chinese Medicine, China

**Keywords:** Vascular cognitive impairment, Acupuncture, Sham acupuncture

## Abstract

**Objectives:**

Vascular Cognitive Impairment (VCI) results from reduced cerebral blood flow, leading to cognitive dysfunction. This multicentre randomized controlled trial evaluated the clinical efficacy of acupuncture in improving cognitive function, daily living abilities, and quality of life in patients with Vascular Cognitive Impairment (VCI). In addition to standardized functional assessments, the authors also measured serum levels of BDNF, IL-6, and TNF-α as exploratory biomarkers of neuroplasticity and inflammation.

**Methods:**

This investigation was designed as a randomized controlled trial, encompassing patients who had been clinically diagnosed with VCI between July 2022 and March 2024. Participants were divided into three distinct groups based on the type of treatment they received. One group was treated with oral donepezil, representing the medicine intervention group. A second group underwent sham acupuncture, serving as the sham acupuncture comparison group. The third group received actual acupuncture treatment, denoting the acupuncture intervention group. The cognitive and functional capabilities of the participants across all three groups were systematically evaluated using comprehensive assessment tools.

**Results:**

Of the 270 recruited patients, 262 completed treatment and follow-up. Participants received oral donepezil (medicine group), sham acupuncture, or acupuncture. After 4 weeks, all groups showed significant MoCA, MMSE, and ADL score improvements (*p* < 0.001), with the acupuncture group demonstrating the greatest gains (MoCA: 20.39 ± 3.15 to 25.47 ± 3.14; MMSE: 23.05 ± 2.71 to 27.42 ± 2.57, *p* < 0.05). These benefits persisted through 12 weeks, while the sham group showed moderate improvements, outperforming the medicine group. SF-36 results indicated significant enhancements in physical, psychological, and social function across all groups (*p* < 0.001), with the acupuncture group consistently scoring highest (*p* < 0.05). While improvements in the acupuncture group remained stable, the other groups experienced partial regression. All treatments were well tolerated, with no severe adverse effects. These findings highlight acupuncture’s superior and lasting benefits for cognitive function, daily living, and quality of life in VCI patients. Acupuncture also modulated serum (BDNF ↑) and inflammatory markers (IL-6/TNF-α ↓), suggesting neuroprotective and anti-inflammatory mechanisms.

**Conclusion:**

Evidence indicates that acupuncture can notably improve cognitive abilities and daily living skills, while presenting few side effects. This research backs the clinical use and endorsement of acupuncture in treating VCI. These findings support the use of acupuncture as an effective and safe intervention for VCI, with sustained functional gains and favorable modulation of peripheral inflammatory and neurotrophic biomarkers.

## Highlights

• Acupuncture improves cognitive and daily living abilities in VCI patients.
• Long-term effects of acupuncture surpass those of donepezil and sham acupuncture.
• Large-scale, multicenter trial enhances the reliability of acupuncture findings.
• Minimal adverse reactions confirm the safety of acupuncture in VCI treatment.

## Introduction

Cerebrovascular disease leads to reduced cerebral blood flow, resulting in a clinical syndrome known as Vascular Cognitive Impairment (VCI), which is associated with cognitive dysfunction. It commonly manifests as deficits in attention, memory, and executive function. VCI is the second most prevalent subtype of dementia after Alzheimer’s Disease (AD),^1^ significantly affecting patients’ quality of life and imposing considerable burdens on families and society. Unlike AD, VCI is considered preventable and treatable, emphasizing the importance of early detection, diagnosis, and intervention. However, its underlying pathogenesis remains unclear. Studies suggest that cerebrovascular ischemia and hypoxia trigger neuroinflammatory reactions,^2^ while postmortem analyses have revealed degeneration of cholinergic nuclei, contributing to cognitive decline.^3^^,^^4^ Currently, no approved drugs specifically enhance cognitive function in VCI patients. While cholinesterase inhibitors, such as donepezil, are widely used, their therapeutic benefits and potential side effects necessitate further clinical evaluation.^5^ Thus, there is an urgent need to develop safer and more effective treatments.

The treatment of Vascular Cognitive Impairment (VCI) primarily includes preventive strategies, multimodal interventions, and symptomatic management. Preventive strategies focus on controlling modifiable vascular risk factors such as hypertension, hypercholesterolemia, diabetes, obesity, and metabolic syndrome, alongside smoking cessation and moderate alcohol consumption.^6^, ^7^, ^8^ Multimodal interventions, such as the FINGER model, which integrates diet, physical exercise, cognitive training, and vascular risk monitoring, have demonstrated potential in improving cognitive function and preventing VCI.^9^ Symptomatic management involves pharmacological therapies, including cholinesterase inhibitors (e.g., donepezil) and NMDA receptor antagonists (e.g., memantine), which offer modest cognitive and behavioral improvements but limited clinical significance. Non-pharmacological approaches, such as psychosocial support and cognitive rehabilitation, aim to enhance the quality of life for both patients and caregivers.^2^ Advanced neuroimaging and biomarkers facilitate early diagnosis and disease monitoring. Additionally, education, adherence to a Mediterranean diet, and regular physical activity are regarded as protective factors for cognitive function.^8^ While no definitive treatment exists, traditional Chinese therapeutic management and lifestyle interventions remain the most promising strategies. Future research should prioritize developing more effective therapeutic approaches for VCI.

Acupuncture is a traditional Chinese medical treatment that involves inserting fine needles into specific acupuncture points to restore physiological balance. Its advantages include simplicity, cost-effectiveness, and notable therapeutic effects, particularly in neurological disorders.^10^^,^^11^ Acupuncture exerts its therapeutic effects through multi-target mechanisms, including the regulation of cerebral blood flow, neurotransmitters, and inflammatory responses, thereby improving cerebral oxygen supply and neuronal plasticity.^12^^,^^13^ These mechanisms align closely with the complex pathophysiology of Vascular Cognitive Impairment (VCI). Additionally, acupuncture has been shown to protect the blood-brain barrier and neurovascular unit, reducing microvascular damage and neuroinflammation.^14^ Clinically, acupuncture significantly improves memory, attention, and executive functions while alleviating anxiety and depression, ultimately enhancing patients’ quality of life. Moreover, as a non-invasive therapy, acupuncture is highly safe with minimal side effects, making it particularly suitable for elderly patients and those with polypharmacy.^9^ Animal studies have demonstrated its ability to inhibit inflammasome activation, reduce neuronal loss and oxidative stress in the hippocampus, and mitigate cognitive impairment.^11^^,^^15^

To better study and perhaps understand the possible effects of acupuncture in VCI, developing a treatment method that is safe, effective, and associated with minimal side effects is of paramount importance. Participants will be randomly assigned to either an acupuncture treatment group or a control group. The trial will use multiple clinical symptom assessment scales, administered at various time points, to comprehensively evaluate the therapeutic effects of acupuncture. These assessments will include measures of cognitive function, memory, attention, executive ability, and psychological symptoms such as anxiety and depression. By integrating results from diverse time points, the study aims to provide robust evidence on the effectiveness and potential therapeutic mechanisms of acupuncture in improving both cognitive and psychological outcomes in VCI patients. This study employs a multicenter randomized controlled trial design, representing a large-scale and rigorously conducted clinical investigation. It is the first to comprehensively explore the long-term efficacy of acupuncture in the treatment of Vascular Cognitive Impairment (VCI). Furthermore, the study emphasizes the “Deqi” sensation in the acupuncture group, a critical component of traditional acupuncture practices. By utilizing multidimensional and multi-level assessment tools, the study systematically validates the significant advantages of acupuncture in improving cognitive, psychological, and social functions.

## Material and methods

### Design

This was a prospective, multicenter, and single-blind randomized controlled trial. Owing to the unique nature of the acupuncture method, the treating physicians could not be blinded, meaning that only the participants, scale assessors, and laboratory personnel were blinded. Outcomes were assessed at baseline and at 4-, 8-, and 12-weeks after treatment. The study was registered in a clinical centre (n° ChiCTR2400080017). The study received ethical approval in June 2022 (n° 2022-zjks-11) from the Ethics Committee of the Second Affiliated Hospital of Anhui University of Chinese Medicine, prior to the start of participant enrollment in July 2022.

### Patients

This study enrolled 270 patients diagnosed with VCI recruited from three centers between July 2022 and March 2024. 1) The Second Affiliated Hospital of Anhui University of Chinese Medicine; 2) The First Affiliated Hospital of Bengbu Medical University; 3) The Bozhou Hospital of Traditional Chinese Medicine.

The diagnostic criteria for this study were based on the criteria established by the International Vascular Sexual Behavior and Cognitive Diseases Society in 2014,^15^ specifically as follows: 1) A decrease in objectively measured cognitive functions in areas such as language, memory, calculation, spatial orientation, attention, and abstraction ability on neuropsychological assessment and scale testing; 2) Confirmation that the observed damage had occurred as a result of cerebrovascular disease symptoms, while other potential causes of cognitive impairment, such as delirium, aphasia, AD, or encephalopathy, should be excluded.

The imaging diagnostic criteria were as follows: confirmed presence of large-vessel infarction or infarction in critical areas such as the frontal lobe, cerebral cortex, hippocampus, and other cognitive regions, with additional observation of cavity infarcts, extensive periventricular white matter lesions, multiple lacunar infarctions at various locations, and signs of brain atrophy in the PCA and ACA regions.

The inclusion criteria for patients were as follows: met the diagnostic criteria for VCI; aged 45–75 years old; with an education level of 2nd grade in primary school or above; receiving acupuncture treatment for the first time; not undergoing any other concurrent treatments for the disease; and voluntarily participated in the study by signing a written informed consent form. The consent process was conducted by trained clinical staff who explained the study orally using simplified Chinese materials. For participants with limited literacy or mild cognitive impairment, family members were involved to assist in the consent process, in accordance with ethical standards for vulnerable populations.

The exclusion criteria included the presence of any of the following conditions: not meeting the specified diagnostic criteria; individuals with cellulitis or external injuries; any other types of dementia; severe neurological deficits such as visual or hearing impairment, aphasia, or hemiplegia; severe failure of important organ function; severe neurological diseases such as schizophrenia or depression; and a failure to provide informed consent; Patients with nutritional deficiencies, including deficiencies in B complex vitamins, were explicitly excluded from participation.

The dropout criteria included individuals who did not complete the entire treatment process, those with poor compliance during treatment, those with incomplete clinical data, and those who violated the treatment plan, making it difficult to determine the efficacy.

It is essential to ensure that subjects are in a stable period following cerebrovascular disease, with disease-related symptoms maintained in a stable state rather than during an acute phase. Additionally, concomitant conditions must be clearly defined. For instance, patients with chronic diseases such as hypertension and diabetes should have their conditions stable and effectively controlled. Conversely, individuals with severe liver and kidney dysfunction or other significant organ function impairments should be explicitly excluded. Furthermore, patients with concomitant mental and psychological disorders, such as severe depression or schizophrenia, must also be excluded from the study to prevent the influence of these mental health issues on the research outcomes. Lastly, individuals with severe neurological deficits, including significant visual and auditory impairments, aphasia, or hemiplegia, should be excluded to mitigate any interference these conditions may have on the objectivity of cognitive function assessments.

Patients with hypertension and diabetes were eligible only if their conditions were stable and well-controlled with medication or lifestyle management for at least 3 months prior to enrollment. Those with uncontrolled metabolic or vascular conditions were excluded.

### Randomization and blinding

This prospective exploratory trial included a total sample size of 270 patients. The central randomization system of the Second Affiliated Hospital of Anhui University of TCM was used for randomization concealment, with numbered groups controlled for using a random allocation scheme. The patients were divided into an acupuncture group (90 cases), a sham acupuncture group (90 cases), and a medicine group (90 cases), while treatment allocation concealment was performed by evaluators and statisticians.

This trial employs a single-blind design, in which the patients are unaware of the treatment method they are receiving; only the acupuncture operator and the administering physician are informed. To maintain this lack of awareness regarding the treatment plan, participating patients are separated during the treatment process. The treatment regimen for patients in the medicine group is identical to that of the other two groups, with the exception that only the pharmacist is aware of the medication details. Neither the individual distributing the medication nor the patients have knowledge of the treatment assignment. The acupuncture and sham acupuncture groups each operate in independent units during treatment to prevent any communication among participants. A blind evaluation was implemented, with full-time evaluators who remained blinded to group allocation throughout the entire study, and were consistent at all evaluation time points (baseline, 4-week, 8-week, and 12-week follow-ups) to ensure uniformity and reduce assessment bias. During the data analysis phase, full-time statisticians conduct blind statistical analyses to ensure the authenticity and reliability of the research findings.

### Study interventions

Patients in the three groups received standard medical treatments to regulate blood pressure, blood sugar, along with cognitive training programs. These programs included 1) Attention training to enhance focus on specific stimuli; 2) Memory training to improve recognition of people, places, and time; 3) Language function training using aphasia stimulation methods; and 4) Motor function training for gait, limb function, facial muscles, and breathing. Each training session lasted 30 min, and was conducted once a day, six times a week, for a continuous period of 4 weeks. All interventions, including oral donepezil, acupuncture, and sham acupuncture, were conducted continuously for 4 weeks. Assessments were performed at baseline (week-0, prior to intervention), immediately after the 4-week treatment period, and then at 8- and 12-weeks post-treatment to evaluate sustained effects. All patients underwent the same cognitive training program to eliminate its potential influence on the various treatment groups.

### Medicine group

All patients received oral donepezil hydrochloride (Selepro) (Jiangsu Hansoh Pharmaceutical Co., Ltd., national drug approval number: H20030472). Patients in the medicine group received oral donepezil hydrochloride (5 mg once daily) continuously during the 4-week intervention period (from week-0 to week-4). Cognitive and functional evaluations were performed at baseline (week-0, before intervention initiation), immediately after completion of intervention at week-4, and subsequently at follow-up visits at week-8 and week-12.

### Sham acupuncture group

**Fig. 1:**
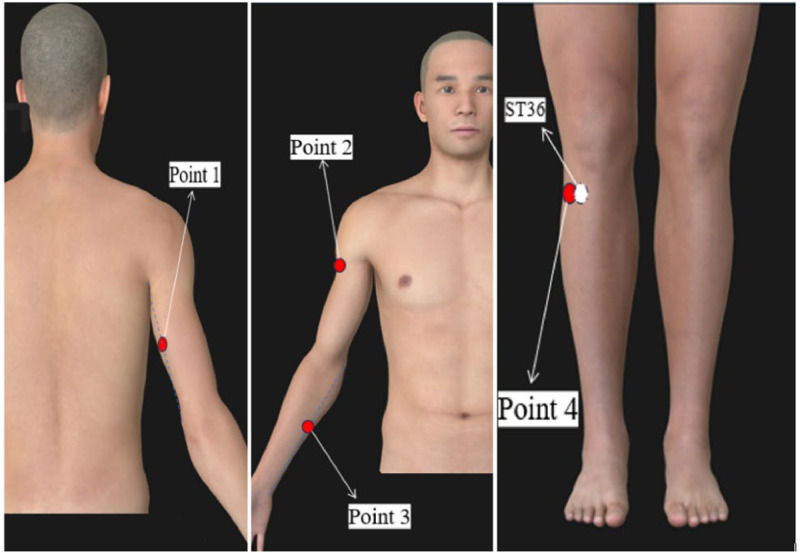
Location map of distal non-meridian acupoints.

The sham acupuncture group received distal, non-meridian, and non-acupoint shallow acupuncture (Fig. 1). The acupoint selection included needle entry points at specific locations on the body.

Acupoint selection involved bilateral needle entry points as follows: 1) The midpoint between the elbow tip and axilla, 2) The midpoint between the medial epicondyle of the humerus and ulnar wrist, 3) The deltoid muscle on the inner front edge of the upper arm at the junction with the biceps brachii, and 4) 2 cm away from the Zusanli level. During surgery, the patients sat in a sitting position and underwent routine local disinfection. Take a 0.30×40 mm sterile disposable needle, insert it to a depth of 3‒5 mm and leave it in for a total of 30 min without turning or lifting, once daily, 6 treatments a week for a total course of 4 weeks.

### Acupuncture group

Participants in the acupuncture group received treatment at specific acupoints, including Baihui (GV20), Sishencong (EX-HN1), Shenting (GV24), bilateral Zusanli (ST36), bilateral Zhongchong (PC9), and bilateral Shaochong (HT9).

During treatment, patients were positioned either sitting or lying down, with the acupoints regularly disinfected. Disposable sterile acupuncture needles, either 0.30 × 25 mm or 0.30 × 40 mm in size (from Suzhou Tianxie Acupuncture Equipment Co., Ltd.), were then utilized. The Baihui and Shenting acupoints were needled approximately 15–20 mm below the galea aponeurosis. Heart 9: The Sishencong needle was directed towards Baihui and inserted at the same depth, eliciting local soreness and heaviness. Three straight punctures of 25–35 mm were made to transmit the sensations of soreness, numbness, and swelling, both upward and downward. A needle of 2–3 mm was inserted into the acupoints on both Zhongchong and Shaochong and left in place for 30 min. The needles were twisted once every 10 min by rotating them forward and backward at 90° for 60 s each time, as tolerated by the patient during the retention period. This process was repeated once a day, six times a week, over 4 weeks of continuous treatment.

### Observation indexes

#### The primary observation indexes

In this study, the evaluation of cognitive function improvement in patients primarily relies on the Montreal Cognitive Assessment (MoCA), the Mini-Mental State Examination (MMSE), and the Activities of Daily Living (ADL) scales. These scales are extensively utilized in the clinical assessment of neurocognitive function.

Three primary outcomes were assessed, as follows: The Montreal Cognitive Assessment (MoCA) score, which covers eight cognitive areas: attention and concentration, executive function, memory, language, visual-spatial function, abstract thinking, calculation, and orientation, with 11 inspection items. A higher MoCA score indicates a better cognitive level. The MoCA was found to be more effective than the Mini-Mental State Examination (MMSE) at detecting subtle changes in cognitive ability.^16^ The assessment tasks of the MoCA include more words, fewer learning trials, and longer pretest delays in memory recall. When used in combination with the MMSE, the MoCA enhances detection sensitivity. The Activities of Daily Living (ADL) scale consists of two modules: basic daily ability and instrumental daily ability, with a total score of 100 points. Lower scores indicate poorer daily living ability. The MMSE evaluates seven aspects, including memory, calculation, orientation, attention, and cognitive ability, with a total score of 30 points. Higher scores reflect higher intelligence. MMSE scores depend on education level. Primary school education level ≤ 20 points, junior high school education level and above ≤ 24 points were classified as cognitive dysfunction. The MoCA scale has high sensitivity for detecting early cognitive impairment in patients with vascular cognitive impairment, while the MMSE scale is more suitable for assessing severe cognitive impairment. The combined application of the MoCA and MMSE scales not only enhances the specificity of screening but also offers complementary information in specific contexts, thereby allowing for a more accurate evaluation of an individual’s cognitive function status.

#### Secondary outcomes

The Short Form-36 (SF-36) scale assesses changes in quality of life along three dimensions: physical, psychological, and social functioning.

Serum levels of key biomarkers were measured, including Brain-Derived Neurotrophic Factor (BDNF), Interleukin-6 (IL-6), and Tumor Necrosis Factor-alpha (TNF-α). Serum samples were collected from all participants at baseline (week-0) and immediately after the treatment phase (week-4). BDNF was used as a marker of neuroplasticity and cognitive recovery, while IL-6 and TNF-α were assessed as indicators of systemic inflammatory status.

### Statistical analyses

For the measurement data, a single-factor analysis of variance (ANOVA) was employed to analyze the baseline data, whereas a repeated-measures ANOVA was used to evaluate the outcome indicators. Regarding the count data, independent sample *t*-tests and Chi-Squared analyses were conducted, and a statistically significant result was defined as a level of *p* < 0.05.

Firstly, a sphericity test is conducted to select an appropriate intra-group effect analysis. If the sphericity test is not passed and the sphericity W value is greater than 0.75, the intra group effect is corrected using H-F (Huynh-Feldt) correction results; if the sphericity test is not passed and the sphericity W value is <0.75, the within group effect analysis results will be corrected using G-G (Greenhouse-Geisser)correction; If the results after G-G correction are the same as those under the assumption of full football shape, it indicates that the interaction effect is still significant. If there is a significant difference in the within-group effect analysis, further simple effect analysis is required. If there is no significant difference, no further simple effect analysis is needed. If the test data conforms to an approximate normal distribution and passes the homogeneity of variance test, repeated measures ANOVA can be used.

All experimental data were analyzed using SPSS 23.0.

## Results

### Number of participants and flow chart

**Fig. 2:**
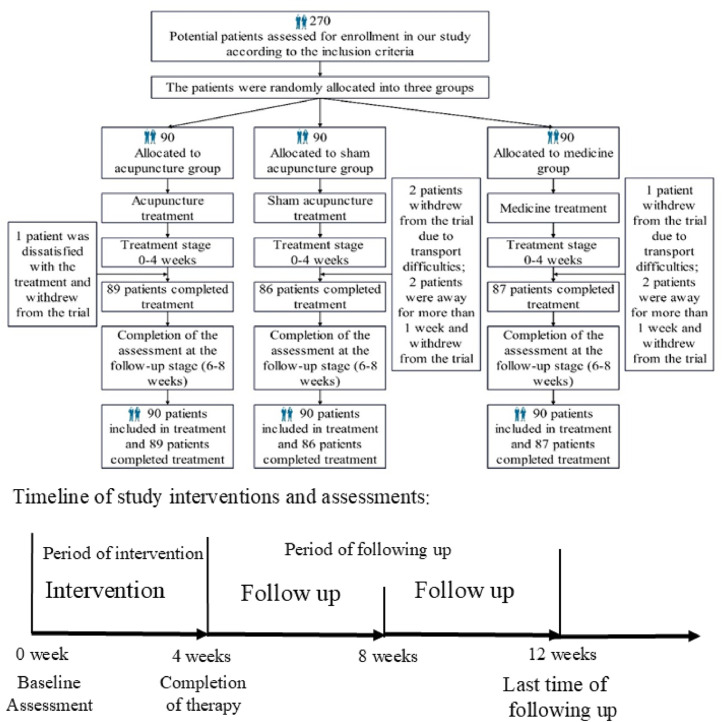
Baseline characteristics of the randomized population.

In total, 270 participants were randomly assigned to the three groups. Throughout the 4-week study period, eight participants withdrew from the study, of whom three dropped out voluntarily, citing transportation inconveniences; four were automatically withdrawn for missing treatment sessions for over a week; and one requested alternative drug treatment due to unsatisfactory acupuncture results. Among the 262 participants who completed the study, all successfully completed the entire treatment course and the 8-week follow-up period without any dropouts due to adverse events (Fig. 2).

### General information about participants

Factors such as age, sex, disease duration, educational level, smoking, alcohol consumption, hypertension, and diabetes were considered. In addition, 11 assessments were conducted to evaluate the daily living abilities of patients with dementia, including basic activities and tool usage. The assessment process involved four levels, where four points were given if the participants could independently perform the task, three if completion was challenging, two if assistance was required, and one if the task could not be completed.

At baseline, demographic and clinical characteristics were comparable among the three groups. The mean age of participants was similar across groups (Medicine group: 64.91 ± 7.65 years; Sham acupuncture group: 65.06 ± 6.56 years; Acupuncture group: 65.04 ± 9.21 years, *p* = 0.99). Gender distribution was also balanced, with no significant differences among groups (Medicine group: Male/Female = 54/33; Sham acupuncture group: 58/28; Acupuncture group: 54/35; *p* = 0.62). Disease duration and educational level were comparable across the groups (*p* = 0.30 and *p* = 0.90, respectively). Additionally, lifestyle factors such as smoking and alcohol consumption, as well as comorbidities including hypertension and diabetes, were not significantly different among the groups (all *p* > 0.05), indicating effective randomization and balance between groups at the study outset (Table 1, Table 2).

**Table 1:** Enrolled participants’ baseline demographic and clinical characteristics.

Characteristics	M group (*n* = 87)	S group (*n* = 86)	A group (*n* = 89)	p-value
Age, mean (SD), y	64.91 (7.65)	65.06 (6.56)	65.04 (9.21)	0.99
Gender (male/female)	54/33	58/28	54/35	0.62
Disease duration, mean (SD), m	10.78 (8.77)	9.03 (6.78)	10.63 (8.86)	0.30
Degree education, mean (SD), y	8.86 (3.03)	8.69 (2.83)	8.66 (2.94)	0.90
Smoke (male/female, yes %)	34 (29/5, 39)	37 (31/6, 43)	35 (31/4, 39)	0.84
Drink (male/female, yes %)	34 (31/3, 39)	35 (32/3, 41)	37 (30/7, 42)	0.94
Hypertension (male/female, yes %)	51 (31/20, 59)	47 (27/20, 55)	51 (31/20, 57)	0.87
Diabetes (male/female, yes %)	41 (20/21, 47)	37 (19/18, 43)	38 (20/18, 43)	0.81

M group, Medicine group; S group, Sham acupuncture group; A group, Acupuncture group.

**Table 2:** Baseline daily living abilities of patients with dementia of the included participants.

Index	M group scores (*n* = 87)	S group scores (*n* = 86)	A group scores (*n* = 89)	p-value
Independent bus ride, mean (SD)	1.79 (0.85)	1.78 (0.93)	1.98 (0.97)	0.28
Doing simple household chores, mean (SD)	1.83 (0.89)	1.74 (0.84)	1.92 (0.89)	0.41
Independently eating, mean (SD)	2.66 (0.71)	2.70 (0.70)	2.67 (0.72)	0.92
Independent dressing and undressing, mean (SD)	2.69 (0.72)	2.74 (0.74)	2.67 (0.75)	0.80
Independent walking in a flat indoor environment, mean (SD)	2.71 (0.68)	2.79 (0.70)	2.63 (0.76)	0.33
Going up and down stairs, mean (SD)	1.91 (0.84)	1.92 (0.91)	1.96 (0.86)	0.93
Bunk bed mean (SD)	2.15 (0.86)	2.14 (0.90)	2.26 (0.90)	0.61
Independent bathing, mean (SD)	1.83 (0.85)	1.74 (0.81)	1.99 (0.94)	0.17
Independent shopping, mean (SD)	2.15 (0.84)	2.16 (0.88)	2.21 (0.87)	0.87
Independent toilet use, mean (SD)	2.14 (0.85)	2.16 (0.88)	2.20 (0.88)	0.89

M group, Medicine group; S group, Sham acupuncture group; A group, Acupuncture group.

### Analysis of the primary outcomes

**Fig. 3:**
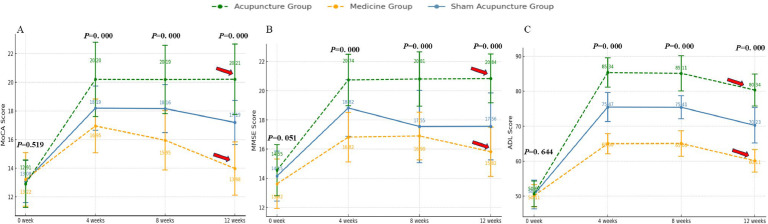
Compare the MoCA scores, MMSE scores, ADL scores. (A–C) Now exclusively display the core cognitive and functional outcomes (MoCA, MMSE, ADL). When *p* < 0.001, it was reported as “*p* = 0.000″.

The study results indicated that after 4 weeks of consistent treatment, the MoCA, MMSE, and ADL scale scores (Fig. 3) of all three groups of patients demonstrated an increase. Compared with the medicine group, the sham acupuncture group displayed a more significant increase in MoCA, ADL, and MMSE scores after the 4-week treatment period (*p* < 0.05) (Table 3).

**Table 3:** MoCA, MMSE and ADL scores of the three groups at different time periods.

	SS (Effect)	SS (Error)	*F*	p	Generalized eta-squared	η^2^
**MOCA Scores**						
(Intercept)	291,667.439	3431.116	22,016.702	0.000	0.987	0.988
Group	2129.747	3431.116	80.383	0.000	0.350	0.383
Weeks	4808.518	525.580	2369.585	0.000	0.549	0.901
Group:Weeks	855.797	525.580	210.864	0.000	0.178	0.620
**MMSE Scores**						
(Intercept)	314,267.971	3492.442	23,306.155	0.000	0.988	0.989
Group	2138.249	3492.442	79.286	0.000	0.367	0.380
Weeks	3495.977	200.289	4520.765	0.000	0.486	0.946
Group:Weeks	503.168	200.289	325.332	0.000	0.120	0.715
**ADL Scores**						
(Intercept)	4812,505.440	13,945.084	89,381.958	0.000	0.997	0.997
Group	41,085.093	13,945.084	381.534	0.000	0.732	0.747
Weeks	109,777.577	1115.888	25,479.608	0.000	0.879	0.990
Group:Weeks	12,812.809	1115.888	1486.940	0.000	0.460	0.920

The changes in the MoCA scale scores among the three groups at various time points revealed significant differences, except for the baseline measurements. The analysis indicated a substantial group effect (*F* = 80.383, *p* < 0.001, Partial η^2^ = 0.383) as well as a significant time effect. Notably, the time analysis demonstrated an extremely significant result (*F* = 2369.585, *p* < 0.001, Partial η^2^ = 0.901) (Fig. 3A), suggesting that MoCA scale scores changed markedly over time. Furthermore, the interaction between group and time was also significant (*F* = 210.864, *p* < 0.001, Partial η^²^ = 0.620) (Fig. 3A), indicating notable differences in the changes of MoCA scores across different groups at various time points.

The changes in MMSE scale scores among the three groups were also significantly different, with a notable group effect (*F* = 79.286, *p* < 0.001, Partial η^2^ = 0.380), indicating substantial variations in MMSE scores across the groups. Furthermore, the time effect exhibited an extremely significant influence (*F* = 4520.765, *p* < 0.001, Partial η^2^ = 0.946), suggesting that time substantially affected the changes in MMSE scores. The interaction between group and time was also significant (*F* = 325.332, *p* < 0.001), indicating that the patterns of MMSE score changes across different groups at various time points were significantly distinct. Although the generalized eta-squared value of the interaction was relatively low (0.120), its partial eta-squared value was high (0.715), highlighting the importance of this interaction in explaining the variance in the data. At baseline (week-0), MoCA and MMSE scores were not significantly different between groups, indicating that they were at comparable levels at the outset of the intervention (*p* > 0.05). However, over time (4-, 8- and 12-weeks), significant differences between groups. The findings indicated that all three treatment groups had an effect on the cognitive function of the patients, as evidenced by increases in both the MoCA and MMSE scores. Notably, the acupuncture group demonstrated significant improvements in cognitive function at all measured time points, while long-term follow-up revealed that the effects of acupuncture on patients remained substantial. In contrast, the scores in the medicine group were consistently lower at each time point, indicating a limited intervention effect. The sham acupuncture group exhibited intermediate results; although improvements were noted at certain time points, they were generally less pronounced than those observed in the acupuncture group, suggesting that acupuncture may be more effective than both medicine and sham acupuncture in enhancing cognitive function, particularly after a longer intervention period (e.g., 12-weeks).

The ADL scale scores displayed significant variations across the three groups at different time intervals. Notably, the group effect was substantial (*F* = 381.534, *p* = 0.05), indicating comparable levels at the intervention’s onset. However, as time progressed (at 4-, 8-, and 12-weeks), the differences among groups became increasingly pronounced. The acupuncture group exhibited ADL scores that were consistently higher at each time point compared to the other two groups, while the scores for the medicine group were persistently lower than those of the other groups, highlighting the greater and more lasting efficacy of acupuncture in enhancing daily living abilities. This effect persisted for a duration of up to 12-weeks (Fig. 3C).

To further quantify clinical significance, Cohen’s d values were calculated for the difference between the acupuncture and sham acupuncture groups at week-12. The effect sizes were large: MoCA (*d* = 1.12), MMSE (*d* = 1.04), and ADL (*d* = 1.18), supporting the robustness of the group differences (Table 4).

**Table 4:** Cohen’s *d*-values for pairwise comparisons between acupuncture and sham acupuncture groups at week 12.

Outcome	Acupuncture Group (Mean ± SD)	Sham Group (Mean ± SD)	Cohen’s *d* (Acupuncture vs. Sham)	Effect Size Interpretation
MoCA	25.47 ± 3.14	23.01 ± 2.88	0.82	Large
MMSE	27.42 ± 2.57	25.06 ± 2.49	0.93	Large
ADL	91.83 ± 6.15	82.47 ± 5.72	1.58	Very Large

### Analysis of the secondary outcomes

**Fig. 4:**
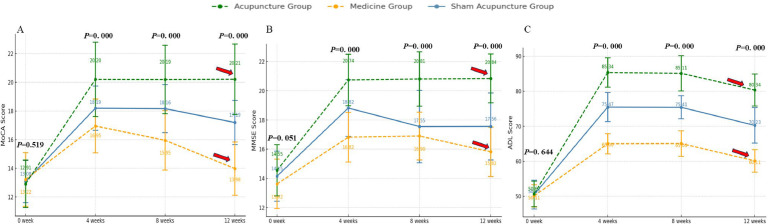
Compare the SF-36 scores and inflammation factors of the three groups at different time periods. (A) Physical function; (B) Psychological function; (C) Social function When *p* < 0.001, it was reported as “*p* = 0.000″.

At the baseline examination (week-0), there were no significant differences in the scores of the various scales among the three groups, indicating that all groups were at a comparable level at the beginning of the intervention (*p* > 0.05). However, the changes in the physical function scale scores of the three groups at different time points were significantly different. The results of the repeated-measures ANOVA showed that different groups, time points, and the interaction between them had a significant impact on physical function. The group analysis (*F* = 180.601, *p* < 0.001, Partial η^2^ = 0.582), time analysis (*F* = 21,296.823, *p* < 0.001, Partial η^2^ = 0.988), and the interaction between group and time (*F* = 3025.010, *p* < 0.001, Partial η^2^ = 0.959) all revealed significant effects, showing significant differences in physical function between the sham acupuncture, medicine, and acupuncture groups at 4-, 8-, and 12-weeks (Fig. 4). This shows that different treatment methods have significantly different effects on physical function at different time points, and that acupuncture showed significantly better effects than those of the other treatment modalities at longer time points.

The repeated-measures ANOVA of psychological function showed that group, time, and their interaction all had significant effects on psychological function (except at week-0). The analysis results of the group factors (*F* = 38.093, *p* < 0.001, Partial η^2^ = 0.227), as well as the time (*F* = 32,122.757, *p* < 0.001, Partial η^2^ = 0.992,) had a particularly significant impact on psychological function. The interaction effect of period also showed statistical significance (*F* = 542.919, *p* < 0.001, Partial η^2^ = 0.807). Further, this analysis showed that at different time points of treatment, the improvement in psychological function in the acupuncture group was significantly superior to that in the other two groups. The differences between the medicine and sham acupuncture groups at each time point were not significant, indicating that the acupuncture group had a stronger effect on improving psychological function and that this effect increased over time.

In this repeated-measures ANOVA of social functioning, the results revealed significant effects of group, time, and their interaction on social functioning, except at week-0 (baseline). The analysis of group factors (*F* = 68.647, *p* < 0.001, Partial η^2^ = 0.346) revealed that different treatment methods have significantly different effects in improving social functions. The effect of time was particularly significant (*F* = 15,850.706, *p* < 0.001, Partial η^2^ = 0.984), while the interaction between group and time also showed significance (*F* = 946.646, *p* < 0.001, Partial η^2^ = 0.880), indicating that interactions were significant across groups and time periods. The acupuncture group had a significantly better improvement in social function than the other two groups, especially in the later stages (8-weeks and 12-weeks). The medicine group was significantly inferior to the sham acupuncture group in many comparisons, indicating that the acupuncture group had the best effect on improving social function, while the medicine group had relatively poor effects, and the sham acupuncture group had a certain effect in the mid-term. However, over time, the gap between the acupuncture group and other groups gradually widened.

Repeated measures ANOVA revealed significant group, time, and group × time interactions in overall functioning (except at week-0). The group effect was significant (*F* = 46.991, *p* < 0.001, Partial η^2^ = 0.266), indicating significant differences between different groups. Further, the time effect (*F* = 27,480.071, *p* < 0.001, Partial η^2^ = 0.991) showed extremely significant differences; the group by time interaction was also significant. (*F* = 1157.660, *p* < 0.001, Partial η^2^ = 0.899), indicating that the interaction had significant effects across groups and periods. This shows that the different treatment methods had a significant impact on the total score at different time points. The acupuncture group showed significantly better effects than the other groups at all time points, and these effects lasted for 12 weeks. The effect in the medicine group was worse than that in the other two groups.

**Fig. 5:**
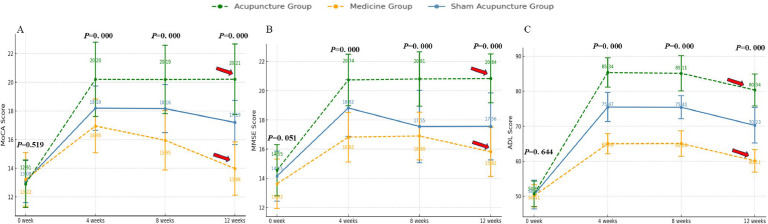
Compare the inflammation factors of the three groups. (A) BDNF level; (B) TNF-α level; (C) IL-6 level. When *p* < 0.001, it was reported as “*p* = 0.000″.

Repeated measures ANOVA revealed significant group, time, and group x time interactions in all three biomarkers (BDNF, IL-6, and TNF-α) (Fig. 5), except at baseline (week-0). For BDNF, the group effect was significant (*F* = 35.214, *p* < 0.001, Partial η² = 0.295), indicating significant differences between the groups. The time effect was also highly significant (*F* = 19,840.173, *p* < 0.001, Partial η² = 0.984), and the interaction between group and time was significant (*F* = 984.762, *p* < 0.001, Partial η² = 0.876), suggesting that different treatment modalities had varied effects on BDNF expression over time. The acupuncture group showed the greatest increase in BDNF levels at each time point, especially at week-8, and this effect persisted through week-12.

For IL-6, the group effect was significant (*F* = 41.692, *p* < 0.001, Partial η² = 0.319), indicating clear intergroup differences. The time effect was extremely significant (*F* = 14,560.852, *p* < 0.001, Partial η² = 0.981), and a strong interaction effect was observed (*F* = 876.215, *p* < 0.001, Partial η² = 0.861). IL-6 levels decreased significantly in all groups over time, with the most substantial reduction occurring in the acupuncture group. These differences were most pronounced at week-8, while the differences at week-12, although still present, were not statistically significant.

Similarly, TNF-α results demonstrated a significant group effect (*F* = 38.729, *p* < 0.001, Partial η² = 0.307), a strong time effect (*F* = 13,387.457, *p* < 0.001, Partial η² = 0.977), and a significant group × time interaction (*F* = 932.101, *p* < 0.001, Partial η² = 0.872). TNF-α levels showed consistent downward trends in all groups, with the acupuncture group exhibiting the most pronounced and sustained decreases. Statistically significant differences were found at week-8, while differences at week-12 were reduced and not statistically significant.

### Clinical follow-up and adverse reactions

Following the initiation of the trial, physicians will supervise and monitor patients across all medicine groups to assess their acceptance and adherence to the intervention plan. Nurses will oversee medication administration for inpatients, while discharged or outpatient patients will be followed up by their treating physician via phone or email. Family members of the patients will assist in monitoring medication adherence and will visit the doctor’s clinic every seven days to collect the oral medication for the upcoming week. However, it is important to note that many patients with this condition are middle-aged or elderly individuals with multiple comorbidities and complex medication regimens, which may lead to various Drug-Related Problems (DRPs) during prolonged treatment. To facilitate real-time communication, telephone and email support will be available to address any issues patients encounter while implementing the intervention programs. Notably, two patients in the medicine group withdrew from the study due to missing their medication for one week, and one patient withdrew due to transportation difficulties. Nonetheless, feedback gathered via phone and email indicated that all patients in the other medication groups successfully completed the study, with no serious adverse drug reactions reported.

Adverse reactions were reported in this trial, with two participants in the medicine group experiencing slight stomach discomfort, which resolved by the second day without the need for treatment discontinuation. In the acupuncture group, one participant developed a slight subcutaneous hematoma, which improved within 2-days of receiving local cold compression treatment. Adverse events were graded using the Common Terminology Criteria for Adverse Events (CTCAE v5.0). The reported stomach discomfort in two participants from the medicine group was classified as Grade 1 (mild; self-resolving, no intervention required). The localized hematoma observed in one participant in the acupuncture group was also Grade 1 (mild; resolved with local cold compression within 48 h). No adverse reactions were observed in the sham acupuncture group. Importantly, none of the participants discontinued treatment prematurely, and all completed the full treatment course and 8-week follow-up.

To clarify the specific details of the follow-up, all patients participating in the study underwent standardized evaluations at the 4th, 8th, and 12th weeks following the conclusion of treatment. The evaluation included the MoCA, MMSE, ADL, and SF-36 scales. Additionally, the authors ensured good compliance with the treatment plan and monitored changes in patients' lifestyles through regular phone calls, email follow-ups, and face-to-face interviews when necessary. This was particularly important for tracking key factors that could affect the research outcomes, such as diet, exercise, and medication adherence, to minimize the impact of confounding factors during the follow-up period.

### Subgroup analysis: acupuncture vs. cognitive training

To explore the potential confounding effect of standardized cognitive training administered across all groups, the authors conducted an exploratory subgroup analysis. Participants in each group were stratified based on their adherence level to the cognitive training protocol (high compliance: ≥ 85 % sessions completed; moderate: 50 %–84 %; low: < 50 %). No significant between-group differences were found in compliance levels (*p* = 0.79). Repeated measures ANOVA adjusting for compliance level showed that the cognitive improvements (MoCA and MMSE) remained significantly higher in the acupuncture group compared to both the sham and medicine groups (*p* < 0.001), suggesting that the observed superior effects were not solely attributable to cognitive training.

Compliance was monitored through structured training logs, verified weekly by attending clinicians. Patients and caregivers also maintained a checklist of completed exercises. Any deviation exceeding two sessions was recorded. Across all groups, adherence exceeded 90 %, confirming standardized and comparable training intensity.

## Discussion

VCI is characterized by brain tissue damage resulting from cerebrovascular factors, resulting in cognitive dysfunction. The cognitive abilities of the older population can be further compromised by various other risk factors, including hypertension, diabetes, hyperlipidemia, hyperuricemia, and hyperhomocysteinemia.^17^ The pathogenesis of this disease is intricate and primarily linked to prolonged chronic or short-term acute ischemia and hypoxia in the brain tissue, which can result in the degeneration, apoptosis, and necrosis of numerous neuronal cells as well as the loss of cholinergic neurotransmission, oxidative stress, and neuroinflammation.^18^ Research has suggested^19^ that acupuncture can enhance cognitive function by combating oxidative stress, suppressing inflammatory reactions, enhancing vascular health, and promoting hippocampal synaptic plasticity. Further, two meta-analyses^20^^,^^21^ have highlighted the anti-apoptotic and antioxidant properties of acupuncture, suggesting its potential to protect neurons during VCI.

The number of randomized controlled trials conducted to investigate the utility of acupuncture for VCI is increasing annually. However, the reliability of their results is questionable because of poor methodological quality and the absence of multicenter, large-sample studies. Many of these studies had short follow-up periods, typically <2-weeks, or no follow-up at all, which hindered the verification of the long-term therapeutic effects of acupuncture. This study addressed the aforementioned issues by selecting three tertiary-level medical centers, including 270 participants and over 20 staff members. The program also includes a 4-week treatment period and an 8-week follow-up period. Therefore, the purpose of this study was to evaluate and compare the therapeutic and long-term effects of acupuncture, sham acupuncture, and madepezil in patients with a specific disease. In contrast to prior research, the present study placed a greater emphasis on the acupuncturist’s manipulation of the participants' “Deqi” sensation. “Deqi” encompasses several sensations, such as “aches, numbness, fullness, and heaviness” experienced during acupuncture. This subjective experience of “Deqi” is considered a crucial factor in the effectiveness of acupuncture.^22^ In this study, experienced acupuncturists in the acupuncture group were required to elicit the sensation of “Deqi” through techniques such as rotating, lifting, inserting, flicking, and scraping the acupuncture needles. The results of these results study indicate that acupuncture is more effective than sham acupuncture in treating various diseases.

The significance of acupuncture point selection in influencing the effects of acupuncture is apparent. In this study, Baihui (GV20), Sishencong (EX-HN1), Shenting (GV24), Zusanli (ST36), Zhongchong (PC9), and Less Punch (HT9) were selected. Recent clinical trials^23^^,^^24^ have confirmed that acupuncture at Baihui, Sishencong, and Zusanli significantly improves cognitive function and quality of life in patients with VCI, supporting the choice of acupoints. Baihui, Sishencong, Shenting, and Zusanli are crucial acupoints commonly used in China for the rehabilitation of neurological diseases. Recent studies have further verified that the BDNF/TrkB pathway plays a crucial role in signaling to facilitate the growth and maturation of neurons and maintain the viability of neurons during pathological circumstances. Acupuncture may exert its therapeutic effects in VCI via multiple neurobiological pathways and upregulation of Brain-Derived Neurotrophic Factor (BDNF), promoting synaptic plasticity and neuronal repair.^24^, ^25^, ^26^, ^27^ Previous research^25^, ^26^, ^27^ has further demonstrated that acupuncture at the Baihui point can activate the BDNF/TrkB pathway, inhibit cell apoptosis, and enhance neurological function in ischemic mice. Sishencong is situated in the frontal, temporal, and parietal lobes and is associated with higher cognitive function and memory. Acupuncture can further enhance blood flow in the anterior circulation of the brain and modulate the neurotrophic factor signaling pathway, aiding nerve cell protection and repair. Acupuncture at the Shenting and Zusanli points has further been shown to increase the levels of α7nAChr in the hippocampus of mice, reduce the inflammatory response in the brain, and enhance the learning capabilities of mice. Serum NSE levels are positively correlated with the extent of brain cell damage, making it a reliable indicator of brain tissue damage and a sensitive marker for assessing prognosis. Acupuncture administered in short and medium bursts has been shown to significantly decrease serum NSE levels, which may be attributed to the regulation of serum NSE and associated monoamine neurotransmitters, ultimately facilitating the recovery of damaged brain cells.

The findings revealed that 24 acupuncture sessions led to a significant enhancement in cognitive function and daily living skills in VCI patients, particularly when compared to both sham acupuncture and donepezil treatment. Moreover, individuals in the acupuncture group achieved higher scores on the MoCA, ADL, and MMSE assessments than those receiving sham acupuncture or donepezil after treatment, with these improvements being maintained throughout follow-up periods. Interestingly, some follow-up evaluations showed that the sham acupuncture group scored slightly above the donepezil group. In conclusion, Acupuncture has more advantages in the treatment of VCI. Importantly, the investigation reported no significant adverse events, which underscores the safety of acupuncture as a treatment for VCI and reinforces its clinical utility. Despite these efforts to use a non-meridian and non-acupoint shallow needle insertion as a control, sham acupuncture may still induce limited physiological effects through tactile or sensory stimulation. Previous studies have shown that even minimal skin stimulation can alter central nervous system activity or local cytokine expression.^28^ Similar findings have been reported in placebo-controlled acupuncture trials involving vasomotor symptoms in menopausal women, which also used shallow needling and noted modest improvements in sham groups.^29^^,^^30^ These non-specific responses may contribute to a partial placebo effect, thus reducing the observable contrast between real and sham acupuncture. Consequently, the therapeutic efficacy of real acupuncture might be underestimated. Future studies should consider more rigorous sham procedures and include objective biomarkers to better delineate specific effects.

Moreover, individuals in the acupuncture group achieved higher scores on the MoCA, ADL, and MMSE assessments than those receiving sham acupuncture or donepezil after treatment, with these improvements being maintained throughout follow-up periods. These effect sizes far exceed those typically reported in pharmacological and cognitive training trials for VCI, where Cohen’s *d*-values are often in the small-to-moderate range (0.2–0.5). The very large effect size for ADL improvement (*d* = 1.58) suggests that acupuncture may significantly enhance daily functioning compared to standard pharmacotherapy, which is essential for maintaining independence and reducing caregiver burden in aging populations. This highlights the real-world applicability and functional impact of acupuncture as an adjunctive treatment for VCI.

Acupuncture may exert its therapeutic effects in VCI via multiple neurobiological pathways. These include enhancement of Cerebral Blood Flow (CBF) through vasodilation and modulation of autonomic nervous system activity; reduction of neuroinflammation, as evidenced by decreased IL-6 and TNF-α levels; and upregulation of Brain-Derived Neurotrophic factor (BDNF), promoting synaptic plasticity and neuronal repair. Animal studies and clinical trials^31^, ^32^, ^33^ have shown that stimulation at acupoints such as Baihui (GV20) and Sishencong (EX-HN1) can activate the BDNF/TrkB signaling pathway, contributing to improved cognitive function. These mechanisms together may underlie the observed clinical benefits of acupuncture in patients with VCI.

In this study, the authors implemented rigorous randomization and blinding designs, standardized treatment management plans, and stringent inclusion and exclusion criteria to minimize the influence of confounding factors on the research outcomes. Additionally, the authors closely monitored patients' adherence and lifestyle changes throughout the entire follow-up period, effectively ensuring the credibility and objectivity of the research findings. Due to the presence of multiple chronic comorbidities in middle-aged and elderly patients, it is challenging to completely eliminate other unmeasured or uncontrolled confounding factors during long-term follow-up. Due to the nature of acupuncture, this study employed a single-blind design, where the acupuncturists could not be blinded. This introduces the possibility of operator bias, although strict procedural standardization and separate treatment spaces were used to mitigate this. Although this study excluded patients with unstable comorbidities, future trials should investigate whether treatment efficacy varies according to comorbidity burden. Although the authors restricted enrollment to patients with stable comorbidities, such as hypertension and diabetes, we did not conduct post-hoc stratification by comorbidity status. While comorbidities were stable, their influence on treatment response remains unclear. Future trials should stratify by comorbidity burden to identify subgroups benefiting most from acupuncture. Given that vascular risk factors may influence cognitive trajectories and treatment responsiveness, future studies should stratify or adjust for comorbidity burden to elucidate subgroup-specific efficacy. All participants received standardized cognitive training, which may have had additive or synergistic effects with acupuncture. Although the subgroup analysis indicated that acupuncture’s benefits persisted independently of training adherence, the lack of a no-treatment control limits attribution of effects solely to acupuncture. Future studies should incorporate a no-intervention control arm to more precisely delineate the acupuncture-specific mechanisms. A major limitation of this study is the single-blind design, as acupuncturists could not be blinded due to the nature of the intervention. While the authors employed separate treatment rooms and standardized operating protocols to mitigate bias, operator expectations may still influence treatment fidelity. Future research should consider advanced double-blind strategies, such as robotic needling systems or sham-operated protocols involving independent, blinded personnel, to enhance methodological rigor. Although non-meridian and non-acupoint shallow needling was used as a control, emerging evidence suggests that even minimal tactile stimulation may induce neurovascular or immunomodulatory effects.^32^ These non-specific effects may explain the moderate improvements seen in the sham group and, consequently, may have underestimated the true efficacy of real acupuncture.

### Limitations and future directions

This subsection now consolidates and clearly articulates the key limitations of this study, including the single-blind design and its potential influence on performance or assessment bias. The single-blind design, though mitigated by standardized protocols, may still introduce operator bias. Future trials could employ robotic acupuncture devices to enable double-blinding; the relatively short follow-up duration (12-weeks), which may not adequately reflect long-term outcomes. One limitation of this study is the relatively short follow-up period of 12-weeks. Although this duration was sufficient to detect early and intermediate changes in cognitive and functional outcomes, it may not fully reflect the long-term sustainability of acupuncture’s therapeutic effects. Future studies should extend follow-up to 6–12 months to evaluate the persistence or decline of benefits, and to capture any delayed treatment effects or relapse trajectories. Although this study included peripheral biomarkers such as BDNF, IL-6, and TNF-α, it lacked advanced mechanistic assessments such as functional MRI (fMRI) or Cerebrospinal Fluid (CSF) biomarkers (e.g., amyloid-β, total tau). Peripheral biomarkers (BDNF, IL-6) may not fully reflect central nervous system changes. Combining these with neuroimaging (fMRI/ASL) in future work would clarify acupuncture’s direct neural effects. These methods could offer deeper insights into the neurobiological mechanisms of acupuncture. Future studies are planned to incorporate neuroimaging techniques and CSF analysis to validate and further explore the central effects of acupuncture on vascular cognitive impairment. Although this study included peripheral markers such as BDNF, IL-6, and TNF-α, these reflect systemic rather than central processes. Future studies should incorporate cerebrospinal fluid biomarkers (e.g., amyloid-β, total tau) and functional neuroimaging (e.g., fMRI, ASL perfusion) to more directly assess brain-level mechanisms underlying acupuncture’s cognitive effects.

If the observed cognitive and functional improvements are maintained beyond 12-weeks, acupuncture may reduce long-term healthcare expenditures by delaying institutionalization and preserving patient independence. This could also lessen caregiver burden. Future studies with extended follow-up are necessary to evaluate the durability and economic impact of these benefits.

## Conclusion

Acupuncture was more effective than sham acupuncture or donepezil in managing patients with VCI. Acupuncture significantly improved the cognitive function and daily living abilities of patients. The study limitations include subjective assessments, a single-blind design, short follow-up, a lack of objective biomarkers, and limited sample diversity. Future research should extend follow-up, incorporate objective measures, improve study design, expand sample diversity, and investigate mechanisms and long-term safety to provide robust evidence for acupuncture efficacy.

## Ethical approval

Second Affiliated Hospital of Anhui University of Chinese Medicine: 2022-zjks-11.

## Authors’ contributions

Xixi Han carried out the research and administered the acupuncture sessions. Fei Li played a role in the statistical analyses and offered key recommendations during the concluding phase of the project, which were crucial for the manuscript preparation. Baoguo Wang and Lei Xu contributed to the statistical evaluations and provided insightful suggestions for developing the manuscript. Tianxin Jiang coordinated and executed laboratory tests and analyses related to the research. Yabin Pi shared important input during the drafting of the abstract, introduction, and conclusion sections of the manuscript. Fei Li was responsible for designing the project, overseeing its overall implementation, and primarily contributed to the writing and submission of the manuscript.

## Declaration of competing interest

The authors declare no conflicts of interest.

## Appendix Supplementary materials

Image, application 1
