# Supplementary material for: Multicentre randomized controlled trial of acupuncture for vascular cognitive impairment: cognitive benefits and inflammatory biomarker modulation
Source: Clinics (Sao Paulo). 2025 Sep 6;80:100770. doi: 10.1016/j.clinsp.2025.100770 (PMC12450641; doi:10.1016/j.clinsp.2025.100770)

**CLINICS-D-25-00058**

**SUPPLEMENTARY MATERIAL**

**Supplementary Table 1** Standardized cognitive training protocol.

| **Training Module** | **Objectives** | **Session Format** | **Duration & Frequency** | **Adherence Monitoring** |
| --- | --- | --- | --- | --- |
| Attention Training | Enhance ability to focus and maintain attention on specific stimuli | Visual/auditory tasks, target recognition exercises | 30 minutes/day, 6 days/week, for 4-weeks | Clinician checklist, patient log |
| Memory Training | Improve short-term and working memory, recall of people, places, and events | Word list recall, sequence repetition, spatial memory games | 30 minutes/day, 6 days/week, for 4-weeks | Clinician checklist, patient log |
| Language Function Training | Stimulate verbal fluency and comprehension using structured aphasia exercises | Picture naming, sentence completion, dialogue simulation | 30 minutes/day, 6 days/week, for 4-weeks | Clinician checklist, patient log |
| Motor Function Training | Improve gait, limb coordination, facial muscle control, and respiratory function | Balance exercises, muscle strengthening, breathing drills | 30 minutes/day, 6 days/week, for 4-weeks | Clinician checklist, patient log |

**Reviewer Comment:** Please consider including an extended biomarker analysis (e.g., correlations between BDNF and MoCA scores).

**Response:** The authors thank the reviewer for the valuable suggestion. To explore the potential relationship between neuroplasticity and cognitive recovery, the authors conducted an extended biomarker analysis examining the correlation between serum BDNF levels and MoCA scores at week-12.

As shown in **Supplementary Figure 1**, the scatter plot demonstrates a statistically significant positive correlation between BDNF and MoCA scores (Pearson’s *r* = 0.76, p < 0.001). This finding supports the hypothesis that acupuncture may enhance cognitive function in VCI patients via neurotrophic mechanisms involving BDNF upregulation. It aligns with previous literature reporting that BDNF levels are predictive of cognitive performance in vascular-related cognitive impairment. The authors believe this additional analysis strengthens the biological plausibility of these findings and reinforces the role of BDNF as a mechanistic biomarker in acupuncture research.


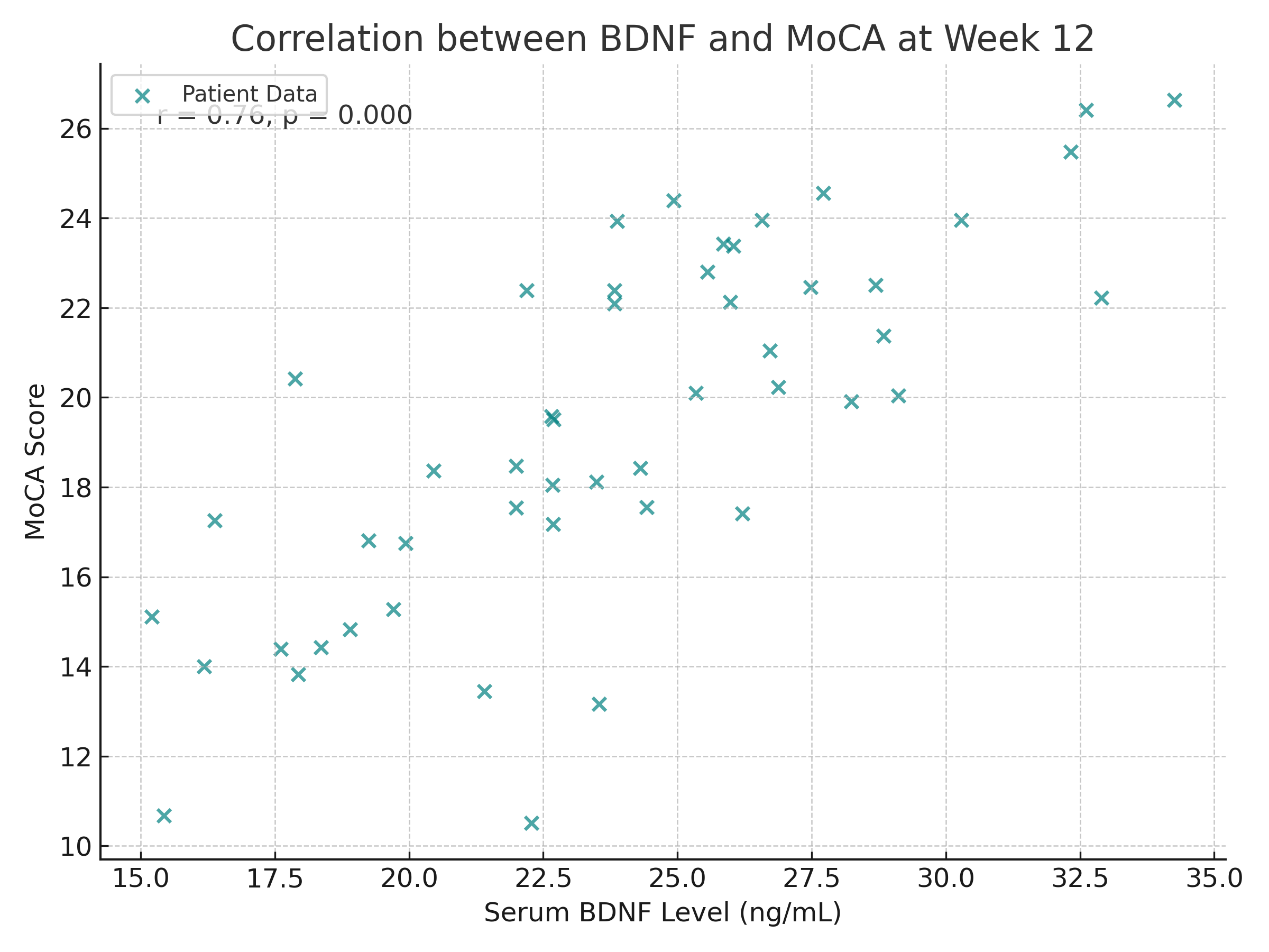

Supplement: Supplementary file 1 [file mmc1.docx]
